# Determinants of Influenza Vaccination Coverage among Greek Health Care Workers amid COVID-19 Pandemic

**DOI:** 10.3390/idr13030071

**Published:** 2021-08-28

**Authors:** Georgios Rachiotis, Dimitrios Papagiannis, Foteini Malli, Ioanna V. Papathanasiou, Ourania Kotsiou, Evangelos C. Fradelos, Zoe Daniil, Konstantinos I. Gourgoulianis

**Affiliations:** 1 Department of Hygiene and Epidemiology, Faculty of Medicine, School of Health Sciences, University of Thessaly, 41222 Larissa, Greece; grach@uth.gr; 2 Public Health & Vaccines Laboratory, Faculty of Nursing, School of Health Sciences, University of Thessaly, 41110 Larissa, Greece; 3 Respiratory Disorders Laboratory, Faculty of Nursing, University of Thessaly, 41110 Larissa, Greece; mallifoteini@yahoo.gr; 4 Community Nursing Laboratory, Faculty of Nursing, University of Thessaly, 41110 Larissa, Greece; iopapathanasiou@yahoo.gr; 5 Faculty of Nursing, University of Thessaly, 41110 Larissa, Greece; raniakotsiou@gmail.com (O.K.); evagelosfradelos@hotmail.com (E.C.F.); 6 Respiratory Medicine Department, Faculty of Medicine, University of Thessaly, 41110 Larissa, Greece; zdaniil@med.uth.gr (Z.D.); kgourg@med.uth.gr (K.I.G.)

**Keywords:** influenza vaccination, health professionals, COVID-19

## Abstract

In the context of the evolving current COVID-19 pandemic, influenza vaccination among the public and health care workers is of particular importance. There are sparse data on the influenza vaccination coverage among health care workers amid COVID-19 pandemic. We aimed to study possible factors associated with influenza vaccination coverage among Greek physicians, dentists, and pharmacists during the flu season 2020–2021. We formulated the hypothesis that COVID-19 pandemic exerted a positive impact on the influenza vaccination coverage of health care workers. An online survey was conducted among the members of the Larissa, (Thessaly, Central Greece) Medical, Dentists, and Pharmacists Associations. The influenza vaccination coverage was 74% (251/340). Acceptance of COVID-19 vaccination coverage was the only factor independently associated with the likelihood of influenza vaccination coverage. In conclusion, we report here a considerable improvement of the influenza vaccination coverage among HCWs and especially among physicians. Acceptance of COVID-19 vaccination was the only predictor of influenza vaccination coverage. This finding provides public health workers and policymakers with useful policy-relevant evidence in order to maintain and even further improve the gains of increased vaccination coverage achieved during the flu season 2020–2021.

## 1. Introduction

According to recommendations from the World Health Organization (WHO) all countries should immunize their healthcare workers in order to protect the vital health infrastructure, and also to control the transmission of influenza to vulnerable, high-risk group of patients [1,2]. In the context of the evolving current COVID-19 pandemic, influenza vaccination among the public and health care workers (HCWs) is of particular importance in order to avoid the overwhelming of the National Health System and drastically reduce respiratory illness related absenteeism among HCWs [1,3]. Influenza vaccination rates among HCWs varies greatly among different countries [4].

There are sparse data on the influenza vaccination coverage among HCWs amid COVID-19 pandemic. There is a study which reported on prevalence of influenza vaccination coverage among a population of physicians, dentists and pharmacists in Central Greece [5]. Nevertheless, in this study determinants of flu vaccination coverage were not investigated. Consequently, using the same dataset, we attempted to study possible factors associated with influenza vaccination coverage (outcome variable) among Greek physicians, dentists, and pharmacists during the flu season 2020–2021. We formulated the hypothesis that COVID-19 pandemic exerted a positive impact on the influenza vaccination coverage of HCWs.

## 2. Methods

Secondary data are being analyzed in the present study. In particular, this survey involved the secondary questionnaire-based data on the COVID-19 vaccination acceptance and associated factors which were obtained from members of the Larissa Medical Association in Central Greece. A comprehensive description of study methodology and data collection was provided previously [5]. In brief, a web-based survey was conducted among the members of the Larissa (Thessaly, Central Greece) Medical, Dentist, and Pharmacist associations, seven days prior to the start of national vaccination campaign against COVID-19 in Greece. The study took place between 15 and 22 December 2020. The members of the above-mentioned associations were invited to complete an online questionnaire. The questionnaire (Table 1) included questions on demographics (sex, age, and profession), attitudes on importance, effectiveness and safety of vaccines. Vaccination coverage against influenza was evaluated with the following item: “Have you been vaccinated with the influenza vaccine (season 2020–2021)”? (Yes/No).

Acceptance and uptake of future COVID-19 vaccination was also evaluated by using the following question: “Are you going to be vaccinated against COVID-19 when the vaccine will be made available?” (Yes/No).

Participants were asked to rate on a four-point Likert scale the importance, safety, and effectiveness of vaccinations (Fully agree/agree/disagree/fully disagree). For the purposes of the logistic regression analysis these four response categories were reduced (Fully agree/agree vs. disagree/fully disagree).

Ethical approval was obtained by the Scientific Committee of the University Hospital of Larissa.

### Statistical Analysis

Absolute (n) and relative frequencies (%) were presented for qualitative data, while quantitative (continuous) data were presented as mean (standard deviation). Chi-square test or Fischer’s exact test was used for the univariate analysis of qualitative variables and Student’s t-test for quantitative (continuous variables. Kolmogorov–Smirnoff test was used for the assessment of normality of continuous data.

Variables found to be statistically significant in the univariate analysis (*p* < 0.25) were included in a stepwise binary logistic regression analysis model, in order to explore potential independent factors associated with influenza vaccination coverage. In this logistic regression model influenza vaccination coverage was the dependent variable and factors found to be significant in univariate analysis were the independent variables. We report results of adjusted odds ratios (ORs) with their 95% confidence intervals (C.I.) for the variables. Statistical analysis was performed by the use of SPSS (SPSS (IBM SPSS Statistics for Windows, Version 22.0., IBM Corp., Armonk, NY, USA), version 22, software.

## 3. Results

In total, 340 (214 physicians, 81 dentists, and 45 pharmacists) HCWs participated in the survey. Among participants. 51% (173/340) were males and 49% (167/340) females. The mean age of the sample was 44.7 years (Standard Deviation = 11; range: 24 to 54 years; skewness = 0.115; SE = 0.132; kyrtosis = −0.513; SE = 0.64).

Table 1 depicts the univariate analysis of influenza vaccination coverage.

Demographic characteristics (sex, age, and occupation) did not exert a significant impact on flu vaccination coverage. On the contrary, belief that vaccines, in general, are important tools for the protection of public health, and that vaccines, in general, are effective against infectious diseases, were significantly associated with the prevalence of influenza vaccination coverage.

This is also the case with COVID-19 vaccination acceptance. HCWs who reported that they will be vaccinated against COVID-19 when the vaccine will become available demonstrated higher influenza vaccination coverage than their counterparts who reported that they are not going to be vaccinated against COVID-19 (78% vs. 59%; *p*-value = 0.001). Three questions were found to be significant in univariate analysis (Vaccines, in general, are important for public health; Vaccines, in general, are effective; have you been vaccinated against COVID-19?) and were included in the logistic regression.

Table 2 presents the results of logistic regression analysis of influenza vaccination coverage. Acceptance of COVID-19 vaccination coverage was the only factor independently associated with the likelihood of influenza vaccination coverage (OR = 2.06; 95% C.I. = 1.15–3.67).

## 4. Discussion

In this descriptive study we examined the correlates of influenza vaccination prevalence among a regional sample of HCWs during the flu season 2020–2021, in the era of the evolving COVID-19 pandemic. We found 74% influenza vaccination coverage. This figure is unpresented and well above previous records of influenza vaccination rates among health care workers in Greece.

Specifically, Rachiotis et al. reported that the acceptance for the 2009 pandemic influenza A (H1N1) vaccine was estimated at 17% (27% among physicians), while Maltezou et al. reported a considerable increase in influenza vaccination coverage among HCWs in Greece over the period 2015–2018 [6,7]. In particular, the vaccination coverage increased from 10.9% to 24.9% among HCWs in hospitals, and from 24.3% to 40.2% among HCWs in primary healthcare centers [7]. Data on HCWs influenza vaccination acceptance and coverage in the context of COVID-19 pandemic are extremely limited. Della Polla et al. found that the flu vaccination coverage among s sample of Italian HCWs during COVID-19 pandemic was 48.1% and previously another Italian study by Di Guiseppe et al. reported a 68% acceptance rate for influenza vaccination [8,9].

Multivariate analysis showed that acceptance of the COVID-19 vaccination (the study was conducted one week prior to the start of the COVID-19 vaccination campaign between 15 and 22 December 2020) was the only factor independently associated with the influenza vaccination coverage (flu season 2020–2021). This interesting point of our multivariate analysis is partially supported by the results from a wide study sample from United Kingdom which found that the COVID-19 pandemic has motivated increased acceptance of influenza vaccination in 2020–2021 in subjects (general population who were previously eligible for the vaccine but routinely unvaccinated [10]. In our survey, acceptance of COVID-19 vaccination would be considered as a surrogate indicator for perceived risk of COVID-19 infection. Further, the finding that coinfection with COVID-19 and influenza increases the risk of death considerably [11] was published prior to the collection of data presented in this survey, and we may speculate that this broadly published finding may have stimulated positive attitudes of the health care workers under study towards influenza vaccination.

Our results should be interpreted with care since there are some limitations which should be taken into account. The regional sample is not entirely representative of the national population of health care workers. However, we believe that the data reported here represent a satisfactory reflection of the intentions of Greek healthcare workers regarding influenza vaccination, given that our sample included participants from Larissa which is a metropolitan city and the capital of the Region of Thessaly which accounts for 8% of the Greek population. It is noteworthy that, in the past, we found in Thessaly an acceptance rate of H1N1 vaccination similar to that provided by a nation-wide study [12]. Nevertheless, future studies with larger samples in hand will validate the findings of the present study. Apart from its regional nature, our sample was very specific since it comprised of physicians, dentists, and pharmacists. Both dentists and pharmacists are health care professionals and as role models for their patients they could influence their vaccine uptake. This is very important for pharmacists especially who are actively engaged in the implementation of the national vaccination program. Furthermore, our questionnaire was short and this may impact on reliability [13]. Last, we acknowledge that our work is based on secondary analysis of COVID-19 vaccination coverage, and flu vaccination coverage was one of the dependent variables. Regarding the questions related to the attitudes towards the vaccines these questions refers to the vaccines in general. By the use of these questions we attempted to explore the attitude of the participants towards vaccination in general. However, the attitudes towards COVID-19 vaccines were investigated by the use of a separate question. Since our study is based on secondary data and the primary outcome of interest was COVID-19 vaccination coverage, we did not directly assess attitudes of the participants towards influenza vaccination. Nevertheless, influenza vaccines had a long history of development and application and could be considered as “old” vaccines. Consequently, we may consider that our questions on the attitudes of participants towards vaccines in general, may indirectly include influenza vaccines too. Our study has policy implications. Reported influenza activity has remained at a very low level throughout the 2020–2021 season, despite widespread and regular testing for influenza viruses [14], possibly due to various reasons, such as wide use of non-pharmaceutical interventions (e.g., face masks, social distancing), increased flu vaccination coverage and competition between influenza and SARS CoV-2. It is difficult to forecast the influenza activity in the future since many uncertainties do exist. However, we cannot exclude the possibility that influenza may show considerable activity during the next season (2021–2022). Increasing immunization coverage is the only way to deal with uncertainties about future influenza activity [15]. Vigilance is needed by public health community and policy makers in order to maintain and even further improve the gains of increased vaccination coverage achieved during the flu season 2020–2021. In conclusion, we report here a considerable improvement of the influenza vaccination coverage among HCWs and especially among physicians. Acceptance of COVID-19 vaccination was the only predictor of influenza vaccination coverage. This finding provides public health workers and policymakers with useful policy-relevant evidence in order to maintain and further increase the influenza vaccination coverage of health care workers.

## Figures and Tables

**Table 1:** Univariate analysis of influenza vaccination coverage (2020–2021).

Variable	Vaccination Coverage		*p*-Value	OR	CI 95%
**Ν % Yes No**					
Sex					
Male Female	132/173 76% 119/167 71%	41/173 24% 48/167 29%	0.3	1.3	0.8–2.1
Age (Mean, Standard Deviation)					
	44.6 ± 11.27	45.2 ± 10.3	0.7		
Overall the vaccines are important for Public Health.					
Fully agree/ Agree Fully disagree/Disagree	250/355 70% 1/5 20%	85/355 30% 4/5 80%	0.018	11.7	1.4–292
Overall the vaccines are safe.					
Fully agree/ Agree Fully disagree/Disagree	244/330 74% 7/10 70%	86/330 26% 3/10 30%	0.72	1.2	0.25–4.7
The vaccines in general are effective.					
Fully agree/ Agree Fully disagree/Disagree	247/329 75% 4/11 36%	82/329 25% 7/11 64%	0.009	5.24	1.5–20.9
Are you going to be vaccinated against COVID-19 when the vaccine will be available?					
Yes No	208/267 78% 43/73 59%	59/267 22% 30/73 41%	0.001	2.45	1.40–4.25
Occupation					
Physicians Dentists/ Pharmacists	162/214 76% 89/126 71%	52/214 24 % 37/126 19 %	0.3	1.29	0.79–2.12

**Table 2:** Logistic regression analysis for influenza vaccination coverage (2020–2021).

Independent Variable	OR	95% C.I.	*p*-Value
COVID-19 vaccination acceptance		1.15–3.67	0.015
Yes	2.06		
No	1.00 (ref)		
Vaccinations are important for the protection of health care workers		0.31–42.3	0.3
Yes	3.61		
No	1.00 (ref)		
Vaccines are effective		0.53–10.29	0.26
Yes	2.34		
No	1.0 (ref)		

## Data Availability

The datasets used and analyzed during the present study are available from the corresponding author on reasonable request.
